# Telemedicine and Utilization of Chest X-Rays in Pediatric Community-Acquired Pneumonia: Lessons from the COVID-19 Lockdown

**DOI:** 10.1177/26924366251394487

**Published:** 2025-11-11

**Authors:** Sophia Eilat-Tsanani, Moti Almog, Vered Levy

**Affiliations:** ^1^ Azrieli Faculty of Medicine, Bar Ilan University, Safed, Israel.; ^2^ Emek Medical Center, Afula, Israel.; ^3^ Family Medicine department, Clalit Health Services, Tel Aviv, Israel.

**Keywords:** chest X-rays, COVID-19, community-acquired pneumonia, pandemic, pediatrics, telemedicine

## Abstract

Community-acquired pneumonia (CAP) is a leading infectious disease in children, with management guidelines emphasizing clinical judgment over routine imaging. The COVID-19 pandemic accelerated telehealth adoption, raising questions about its impact on pediatric CAP care. We conducted a retrospective database study within primary care, comparing chest X-ray use for CAP diagnosis during three lockdown periods (2020–2021) with corresponding pre-pandemic periods (2018–2019). Children aged 1–14 years with CAP diagnoses were included (*n* = 3,499 pre-pandemic; *n* = 438 during lockdown). Remote consultations increased significantly (1.7% vs. 11.6%, *p* < 0.001), while chest X-ray referrals remained stable, and antibiotic use also stayed consistent. Predictors for imaging shifted: pre-pandemic, older age and longer travel distance were significant factors, whereas during lockdown, only higher socioeconomic status was associated. The ongoing use of telemedicine after COVID-19 offers an opportunity to develop strategies that improve diagnostic confidence and encourage “choosing wisely” in pediatric pneumonia diagnosis.

## Background

Community-acquired pneumonia (CAP) is a common infectious disease in pediatric primary care. Viruses are the most common causes of CAP, especially in children before school age. Evidence-based guidelines for pediatric CAP management primarily rely on clinical judgment. These guidelines support diagnosis based on physical examination without the routine use of chest X-rays or laboratory tests, which are often unavailable in community settings.^1,2^ Similarly, antibiotic treatment is recommended selectively, based on clinical signs indicating more severe illness.^1–3^ However, relying solely on clinical findings can make diagnosing CAP difficult and has been linked to inappropriate antibiotic use.^4,5^

The COVID-19 pandemic greatly changed health care delivery. Social distancing measures accelerated the adoption of telemedicine in primary care, including pediatrics.^6^

During the pandemic, the use of telemedicine increased and evolved, aimed at responding to the need for remote consultations in a safe environment. Synchronous visits via telephone or video helped bridge gaps, offering access regardless of patients’ social status or distance from medical centers.^7^ In a policy statement by the American Academy of Pediatrics in 2021, the infrastructure needs for telehealth were discussed extensively.^7^

Telehealth services persist after the COVID-19 pandemic, creating the need to evaluate this new mode of care, as reflected in The Lancet initiative to establish a commission to conclude from the pandemic period.^8^

Regarding the clinical content of remote visits, both clinicians and parents agree that while virtual visits improved access, they were limited by the lack of physical examinations.^9^

Caring for a child with CAP during lockdown challenged health care providers and parents amid stress and uncertainty, which could influence the use of remote consultations and additional referrals for chest X-rays.

We aimed to examine the use of chest X-rays in diagnosing pediatric CAP during the early COVID-19 pandemic in Israel, when citizens were under lockdown, compared to the previous period.

## Methods

We conducted a retrospective database study including children aged 1–14 years with documented visits with a diagnosis of pneumonia during the lockdown period and the corresponding period in the previous year. We excluded children referred to the hospital, those hospitalized within 1 month prior to the index visit, children diagnosed with pneumonia in the previous month, and children diagnosed with an additional infectious disease at the index visit. Additionally, institutionalized children and those with disabilities, immunodeficiency, or cystic fibrosis were also excluded.

Clalit Health Services (CHS) is the largest health care provider in Israel, operating under the National Health Law and offering nationwide hospital and community-based services. Primary care visits and blood tests are provided at no charge; imaging requires a minimal co-payment, and medications are available at subsidized prices. Before the COVID-19 pandemic, telehealth services had already been introduced at CHS, but became widely used during the pandemic.

Primary care pediatricians manage remote consultations by telephone in the practice while documenting the visit in the medical record, identifiable in the mode of visit field. Parents choose the mode of visits when making an appointment, but doctors can ask parents to come to the clinic if they find it more suitable. During the pandemic, children could be examined in the practice according to instructions aligned with lockdown restrictions. Primary care doctors at CHS are paid per capita, and their salaries were not affected by the number or mode of visits during the pandemic.

We compared data from three pandemic lockdown periods in Israel (March 25–May 4, 2020; September 18–October 17, 2020; and December 27, 2020–February 7, 2021) (the lockdown period) to the corresponding calendar periods in the pre-pandemic years (2018–2019) (the pre-pandemic period).

**FIG. 1.**
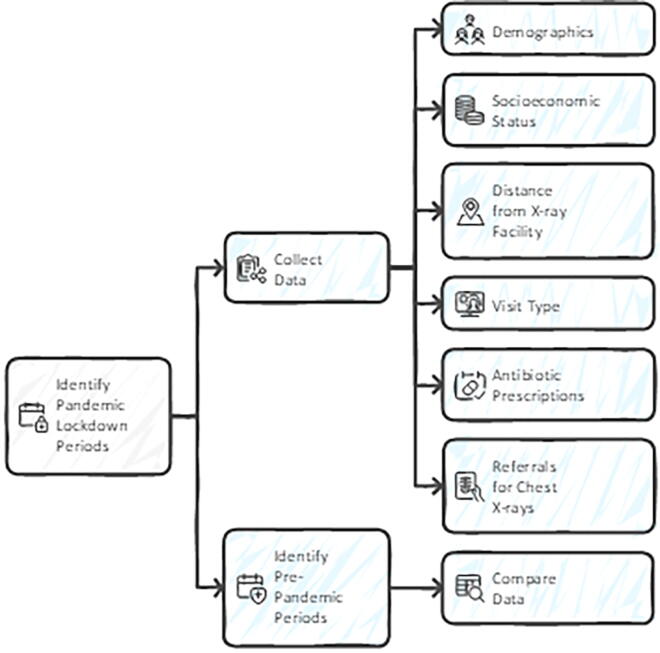
Data collection process. We compared data from three pandemic lockdown periods in Israel (March 25–May 4, 2020; September 18–October 17, 2020; and December 27, 2020–February 7, 2021) to corresponding calendar periods in the pre-pandemic years (2018–2019). Collected data included demographics—age, sex, ethnicity (Jewish or Arab), socioeconomic status (SES), distance of living from X-rays facility, visit type (in-person or virtual), antibiotic prescriptions, and referrals for chest X-rays during the week following the index visit.

Collected data included demographics—age, sex, socioeconomic status (SES), distance from X-ray facilities, visit type (in-person or virtual), antibiotic prescriptions, and referrals for chest X-rays within the week following the index visit (Fig. 1).

### Statistical analysis

The data were analyzed using SAS version 9.4, and *p* < 0.05 was considered significant. Categorical data were reported as number (%), and associations were performed using the chi-square test. Age was reported as mean ± SD, and between-group comparisons were performed using *t*-test. Logistic regression analysis was used to predict referrals for X-rays within a week.

Ethical approval was obtained from the CHS review board (COM1-0061-23).

## Results

The study population included 3499 children who visited their doctors’ clinics with pneumonia diagnoses during the pre-pandemic period and 438 during the lockdown period. The rate of remote visits in children with a diagnosis of pneumonia increased during the lockdown period (from 1.7% to 11.6%, *p* < 0.001). The visits during the lockdown period were characterized by a higher rate of younger children, aged 1–5 years (83.1% vs. 77.9%, *p* = 0.01), and a higher rate of boys (58.9% vs. 53%, *p* = 0.02). (Table 1).

**Table 1.** Characteristics of the Visiting Children Comparing of the COVID-19 Lockdown Period and the Corresponding Period in the Pre-Pandemic Years

Characteristics of the participants	Pre-COVID-19 pandemic period *N* = 3499 *N* (%)	The COVID-19 lockdown period *N* = 438 *N* (%)	*p* value
Age, mean ± std	3.8 ± 3.2	3.3 ± 2.9	<0.001
Age			
1–5 years	2727 (77.9)	364 (83.1)	0.013
6–14 years	772 (22.1)	74 (16.9)	
Sex (boys)	1856 (53.0)	258 (58.9)	0.020
SES, clinic			
Low	2326 (66.5)	297 (67.8)	0.577
Medium-high	1173 (33.5)	141 (32.2)	
Travel time to the Radiology facility			0.545
>30 min	209 (6.0)	23 (5.3)	0.545
≤30 min	3,290 (94.0)	415 (94.7)	
Type of visit			
Remote	59 (1.7)	51 (11.6)	<0.001
Frontal	3438 (98.3)	387 (88.4)	
Doctors’ activities in the visits			
Rreferral to X-rays within 7 days from the visit date	571 (16.3)	81 (18.5)	0.249
Antibiotic prescription within 7 days from the visit date	1997 (57.1)	253 (57.8)	0.784

Approximately two-thirds of the children diagnosed with pneumonia were referred for X-ray examinations, and the same rate were prescribed antibiotics, without change between periods.

Comparing the characteristics of the children who were referred to chest X-rays and those who were not in both periods reveals some differences (Table 2). In the pre-pandemic period, the rate of older children (6–14 years) was higher (30.3% vs. 20.5%, *p* < 0.01), but not in the lockdown period. Living at a distance from the radiology facility of longer than a 30 min drive was at a higher rate only in the pre-pandemic period (9.6% vs. 5.3%, *p* < 0.001).

**Table 2.** Characteristics of the Children Who Were Referred to Chest X-Rays Comparing of the COVID-19 Lockdown Period and the Corresponding Period in the Pre-Pandemic Years

	Pre-COVID-19 pandemic period *N* = 3499			The COVID-19 lockdown period *N* = 438		
	Referral to X-rays within 7 days from the visit date		*p* value^1^	Referral to X-rays within 7 days from the visit date		*p* value^1^
	No *N* = 2928	Yes *N* = 571		No *N* = 357	Yes *N* = 81	
Sex						
Boys	1544 (52.7)	312 (54.6)	0.403	211 (59.1)	47 (58.0)	0.859
Girls	1384 (47.3)	259 (45.4)		146 (40.9)	34 (42.0)	
Age						
1–5 years	2329 (79.5)	398 (69.7)	<0.001	299 (83.8)	65 (80.3)	0.447
6–14 years	599 (20.5)	173 (30.3)		58 (16.3)	16 (19.7)	
Visit type						
Remote	47 (1.6)	12 (2.1)	0.393	42 (11.8)	9 (11.1)	0.869
Frontal	2881 (98.4)	557 (97.9)		315 (88.2)	72 (88.9)	
SES						
Low	1967 (67.2)	359 (62.9)	0.046	262 (73.4)	35 (43.2)	<0.001
Medium/High	961 (32.8)	212 (37.1)		95 (26.61)	46 (56.8)	
Travel time to the radiology facility						
≤30 min	2774 (94.7)	516 (90.4)	<0.001	339 (95.0)	76 (93.8)	0.593
>30 min	154 (5.3)	55 (9.6)		18 (5.0)	5 (6.2)	

^1^ Chi-square test or Fisher’s exact test for categorical variables and T-test for age (continues)

In the multi regression analysis (Table 3) we found that factors predicting referral to chest X-rays were different between the periods. A higher SES could predict referral only in the lockdown period (OR = 4.10; 95% CI: 2.43–6.91), while older age (OR = 1.687; 95% CI: 1.37–2.05) and driving distance longer than 30 min predicted referral to chest X-rays only in the pre-pandemic period (OR = 1.83; 95% CI: 1.30–2.59). The mode of visit, frontal or remote, did not affect the prediction of referral to chest X-rays.

**Table 3.** Logistic Regression Analysis for the Prediction of Referral for X-Rays within a Week

	2018	2020
Sex (Girls vs. Boys)	0.92 [0.77–1.10]	1.03 [0.62–1.72]
Age (6–14 years vs. 1–5 years)	**1.68 [1.37–2.05]**	1.35 [0.71–2.57]
Visit type (Frontal vs. Remote)	0.80 [0.42–1.54]	1.32 [0.59–2.93]
SES (Medium/High vs. Low)	1.08 [0.88–1.32]	**4.10 [2.43–6.91]**
Distance (>0.5 h vs. <0.5 h)	**1.83 [1.30–2.59]**	0.49 [0.17–1.44]

Bold values indicate factors predicting referral to chest X-rays.

## Discussion

In our study, the increase in the rate of remote visits for children diagnosed with pneumonia during the lockdown period was not associated with an increase in referrals for chest X-rays. Similarly, the use of antibiotics remained unchanged.

When comparing visits between the periods, despite a relatively higher rate of remote visits, frontal visits during the lockdown remained predominant. The rate of younger children cared for during the pandemic increased. The number of boys increased slightly, supporting the observation that boys are more affected by CAP than girls.^10^

Variables associated with referrals for chest X-rays differed: pre-pandemic, older age and longer travel distances were associated, whereas during the lockdown, only higher SES was linked.

The widespread use of imaging and antibiotics, not in accordance with guidelines,^1,2^ was the same in both periods. Together, this may reflect the challenges of making decisions amid uncertainty, as noted in previous studies.^4,5^ Sha et al. addressed this issue in their systematic review on the accuracy of symptoms and physical exam findings for identifying children with radiographical pneumonia.^11^ They identified moderate hypoxemia and difficulty breathing as the most associated with pneumonia.

The unexpected conditions caused by the COVID-19 pandemic have presented opportunities to strengthen child care through telehealth services, ensuring equity among children from diverse backgrounds.^7^ The initiatives developed during this time still persist and maintain better continuity of care between hospitals and homes as well as higher accessibility of specialist care.^12^ This has also led to efforts aimed at enhancing telehealth care quality, such as promoting appropriate antibiotic prescribing during remote visits.^13^

A limitation stems from the source of data in medical records, relying on doctors’ documentation, which could introduce selection bias. Additionally, we could not track the decision-making process behind choosing the type of visit—whether initiated by parents or requested by the doctor after the initial call. However, the availability of detailed data enabling follow-up after health care activities is a strength of our study. The fact that remote visits did not impact doctors’ income rules out financial motives as a factor.

The growing adoption of remote visits marks an irreversible shift, offering benefits during crises, in remote areas, and for parental convenience. Building on Sha et al.’s conclusion that physical signs like moderate hypoxemia and difficulty breathing contribute to pneumonia diagnosis,^11^ we suggest using home pulse oximeters during video consultations. This could improve assessment accuracy and support clinicians in decision-making.

## Conclusions

The challenges imposed by the COVID-19 pandemic serve as a milestone to develop strategies that improve diagnostic confidence and encourage “choosing wisely” in pediatric pneumonia diagnosis.

## Data Availability

The datasets presented in this article are not readily available because the study was conducted in the MDClone system. Since that, data are unavailable for use by other researchers. Further inquiries should be directed to the corresponding author.

## Abbreviations Used

CAP: Community-acquired pneumonia
CHS: Clalit Health Services
SES: Socioeconomic status
